# Special Issue: “Exercise Intervention during Pregnancy and Maternal Health”

**DOI:** 10.3390/jcm11113108

**Published:** 2022-05-31

**Authors:** Rubén Barakat, Ignacio Refoyo

**Affiliations:** 1 AFIPE Research Group, Universidad Politécnica de Madrid, 28040 Madrid, Spain; barakatruben@gmail.com; 2 Sports Department, Universidad Politécnica de Madrid, 28040 Madrid, Spain

## 1. Pregnancy, a Unique and Complex Process

For nine months, the process of pregnancy modifies all the organs and systems of the woman’s body in order to achieve adequate fetal growth and development. This can determine the health of the mother and child later in life. Efficient work and adaptation of all the physiological, mental, and emotional aspects involved are essential. Complications in any of these elements and their corresponding pathologies could have an impact on the mother, the fetus, and the newborn [1,2].

In this sense, scientific evidence confirms the negative consequences of current unhealthy lifestyles in relation to pregnancy outcomes that include the mother, the newborn, and the infant. Leading an unhealthy lifestyle during pregnancy increases the risk of chronic diseases for both mother and fetus [3,4]. In this sense, the worldwide epidemic of obesity and sedentary lifestyle adversely affects pregnancy and postnatal outcomes [5].

## 2. COVID-19—Risks Associated with an Inactive Pregnancy

The impact of COVID-19 has generated a global crisis never before experienced, which affects physiological, emotional, mental, and social factors for all population groups, including those who are pregnant with important related risks [6]. The complications associated with confinement (no group support, reduced mobility, distance between people, etc.) significantly affect the lifestyle of pregnant individuals [7] and potentially remove one of the basic recommendations established by the international scientific community: a physically active lifestyle [8,9].

Due to the nature of the gestational period, adopting an unhealthy lifestyle could result in chronic diseases for the mother and offspring beyond pregnancy, as confirmed by recent scientific evidence [10,11]. Unfortunately, the restrictions resulting from the COVID-19 pandemic (reduced mobility and social contacts) may lead to an increase in sedentary behavior for the pregnant population, resulting in complex consequences in the future [12].

## 3. Unhealthy Lifestyle: Maternal and Fetal Complications

One of the most worrisome adverse outcomes of an unhealthy lifestyle during pregnancy is excessive maternal weight gain, which extends its consequences beyond childbirth with metabolic, cardiovascular, and emotional disturbances [13,14]. Regarding perinatal outcomes, the duration and type of delivery are other factors affected by an unhealthy lifestyle. Longer, more instrumental deliveries and a higher prevalence of cesarean sections are associated with unhealthy lifestyles [15,16].

Another pathology of increasing prevalence worldwide is gestational diabetes mellitus (GDM), with recent data reporting an increasing global prevalence. GDM is directly associated with maternal, fetal, and newborn complications. Some epigenetic trend studies warn about the large number and variety of risks of an adverse intrauterine metabolic environment (such as GDM) with chronic diseases in adult life [17,18,19,20].

An unhealthy lifestyle during pregnancy is also linked to the development of gestational hypertension (GH). In this sense, pregnancies associated with severe GH have an increased risk of maternal and perinatal morbidity. These pregnancies have rates of preterm delivery, small for gestational age (SGA) infants, and abruption placentae significantly higher than the rates in the general obstetrical population and similar to rates reported for women with severe features of preeclampsia [21,22]. GH is associated with the development of hypertension later in life and also with the development of diseases related to hypertension (cardiovascular disease, hyperlipidemia, chronic kidney disease, diabetes mellitus) [23].

## 4. Childhood Obesity

It is interesting to observe that, according to research carried out in recent years, one of the most significant effects caused by excessive gestational weight gain and uncontrolled metabolic environment is the development of childhood obesity. As an epidemic of increasing prevalence with a difficult solution, both worldwide and in Spain, it is imperative that strategies be developed to attempt to curb this outbreak. It is estimated that 43 million children, 22 million of them under the age of 5, are overweight or obese, and that 1 in 3 adolescents have excessive body weight. If this trend continues, the prevalence may reach 9% or 60 million people worldwide in the near future [24,25,26].

Many studies confirm that pregnancy may be a determining factor in this epidemic. Starting pregnancy with a high Body Mass Index and also gaining excessive weight during pregnancy are associated with various maternal–fetal complications. The situation of obesity or overweight during pregnancy is related to a greater predisposition to developing preeclampsia, abortion, cesarean section or instrumental delivery, neonatal death, fetal macrosomia, and metabolic disorders in the fetus. In addition, important postnatal complications may occur, such as excessive retention of maternal weight and metabolic disorders in the later life of the mother and child [27,28,29].

## 5. Mental and Emotional Status during Pregnancy

From a psychological and emotional point of view, pregnancy is a very unstable period, with phases of great lability that expose the woman to the risk of complications and pathologies such as prenatal stress, anxiety, or depression, which could be responsible for complications in the newborn [30]. Sadness, decreased interest in daily activities, and reduced energy and concentration are some of the symptoms that may appear early in pregnancy. Later in pregnancy, there is the possibility of feelings such as being overwhelmed, uneasiness, threat or imminent danger, uncertainty, difficulty making decisions, or obsessive thoughts [31]

Many studies show that gestational psychological and emotional complications influence maternal, fetal, newborn, and even infant wellbeing. The scientific literature reports adverse outcomes such as premature delivery, longer and instrumental deliveries, low birth weight, and even alterations in the physical and cognitive development of the baby, as well as in the mother–child relationship [32,33,34].

Prior to the COVID-19 pandemic, data on the prevalence of gestational psychological and emotional disorders reported values between 15 and 30%. Unfortunately, the complex social and economic situation caused by the pandemic has raised these values; currently, the rate of prenatal depression is at 37%, and 57% of pregnant women report anxiety symptoms during pregnancy [35,36].

Regarding the treatment of mental and emotional complications during pregnancy, an important limitation exists: the impossibility of using pharmacology, which limits the intervention and generates the need for new therapeutic and preventive alternatives [37,38].

Obviously, the COVID-19 pandemic has affected the mental and emotional health (and other factors) of the pregnant population, which calls for new studies in order to find new preventive and therapeutic policies. Exercise could be an interesting option [39].

## 6. Recommendations of Exercise during Pregnancy

There are important recommendations promoted by the scientific community about establishing elements that ensure a healthy pregnancy [40]. However, a small percentage of pregnant women meet these recommendations [8]. In fact, the scientific literature reports an increase in the risk factors of pregnancy disorders associated with an unhealthy lifestyle; for example, a high percentage of women continue to gain excessive weight during pregnancy in spite of mentioned health recommendations, thus increasing the possibility of complications and associated pathologies [19].

Human, clinical, and material resources therefore must be available for the appropriate treatment. There are no official estimates of healthcare costs that these complications generate, but obviously, large budgets could be required.

## 7. Physical Exercise as a Preventive Factor

Efficient preventive tools that collaborate to improve the wellbeing of the pregnant woman and her offspring in a non-invasive way are urgently needed. Physical exercise throughout pregnancy does not alter maternal and fetal parameters and even generates improvements in many pre-, peri-, and postnatal pregnancy outcomes, which have been scientifically proven over the last 30 years [41,42].
